# Food Neophobia, Familiarity with French Cuisine, Body Mass, and Restaurant Food Choices in a Sample of Polish Women

**DOI:** 10.3390/nu14071502

**Published:** 2022-04-03

**Authors:** Dominika Guzek, Dominika Głąbska

**Affiliations:** 1 Department of Food Market and Consumer Research, Institute of Human Nutrition Sciences, Warsaw University of Life Sciences (SGGW-WULS), 02-776 Warsaw, Poland; 2 Department of Dietetics, Institute of Human Nutrition Sciences, Warsaw University of Life Sciences (SGGW-WULS), 02-776 Warsaw, Poland; dominika_glabska@sggw.edu.pl

**Keywords:** food neophobia scale (FNS), food neophobia, French cuisine, menu, ingredients, familiarity, body mass, body mass index (BMI), food choice, consumer

## Abstract

Food neophobia, a condition characterized by a reluctance or avoidance of unknown foods and meals, may influence food choice, and is also associated with body mass and familiarity with food items. This study aimed to analyze the associations between food neophobia, familiarity with French cuisine, body mass, and French restaurant menu food choices in a sample of 203 young Polish women. The Computer-Assisted Web Interview (CAWI) method was used in the study. The food choice questionnaire used for assessment was based on a model French restaurant menu, with dishes planned using a 2 × 2 factorial design for the components of neophobic potential (unfamiliar to Polish consumers) and animal-based components. Food neophobia, familiarity with French cuisine, and body mass were considered independent variables. The food neophobia scale (FNS) developed by Pliner and Hobden was used to assess food neophobia among respondents. The results showed an association between food neophobia and familiarity with French cuisine and French restaurant menu food choices (*p* ≤ 0.05), but no association with body mass was observed (*p* > 0.05). The respondents with a high level of food neophobia chose dishes with neophobic components (for soups and desserts) less often compared to those with a low neophobia level, and in the absence of such an association, they chose dishes with animal-based components (for starters and main courses) less often (*p* ≤ 0.05). The respondents who declared that they were familiar with French cuisine chose dishes with animal-based components (for starters and desserts) more often than those with no familiarity, but a reverse association was observed for soups (*p* ≤ 0.05). Based on the findings of the study, it may be concluded that food neophobia and familiarity with French cuisine may be important determinants of food choice within a French restaurant menu. The study did not show any association between body mass and the choice of dishes from the model French restaurant menu. The findings suggest that the presence of unfamiliar and animal-based ingredients may reduce the frequency of choosing specific dishes within a French restaurant menu, which may reduce the diversity of individuals’ diets.

## 1. Introduction

Restaurant dining has become an important part of life, and except for having a simple, quick, and convenient meal, dining out has become increasingly associated with social life, business, and celebrations [1]. Depending on the reason for restaurant dining, the choice of restaurant [2], as well as meal [3], is determined by various factors. During the current global coronavirus disease 19 (COVID-19) pandemic [4], which has significantly influenced nutritional behaviors and altered the food preferences and food choice determinants [5] of individuals, the ban on indoor dining in restaurants greatly affected social life [6]. However, dining out was partially replaced by online food ordering from restaurants, which has also been linked with specific meal choice determinants [7].

Food choice determinants can be classified within different groups of factors associated with health, emotions, economic aspects and availability, social and cultural aspects, environment and political aspects, as well as marketing and commercial aspects [8]. However, the determinants of meal choice in terms of restaurant menus may differ from general food choice determinants [9]. The dominant determinants may result from the type of restaurant [2] and the cuisine offered, as indicated by Bell et al. [10], who found that branding a restaurant as Italian increased the choice of pasta meals and desserts in that restaurant.

In the case of national cuisine or ethnic cuisine restaurants, food neophobia is an important factor influencing meal choice [11]. Food neophobia is defined as a reluctance or avoidance of unknown food products and meals [12]. It is observed especially among children [13], but according to recent studies, this condition can also be observed in adults, leading to a decreased consumption of or preference for specific foods [14], which constitutes a barrier to dietary change and the management of diet-related health problems [15].

Previous studies have shown that food neophobia seemed to influence food choices in the Polish population, both in Italian restaurants (highly familiar to Polish consumers) [16] and Vietnamese restaurants (relatively unknown to Polish consumers) [17]. However, familiarity with a specific cuisine may be another important determinant of restaurant meal choice, as familiar food products are generally preferred over unfamiliar ones [18]. Moreover, because specific emotions toward animal-based products may interfere with food choices, the willingness to try unknown food products with and without animal-based components may differ [19].

As described above, food neophobia is associated with the choice of food products, including the choice of dishes on a restaurant menu [16,17,18]. At the same time, food neophobia has been linked with body mass, as stated for children in a systematic review and meta-analysis by Cole et al. [20], but also for adults [21]. The choice of food products, including the choice of restaurant dishes, may also be associated with body mass [22], which may be due to the energy value of dishes, as meals of a high energy value may contribute to excessive body mass [23]. 

Considering the abovementioned potential determinants of restaurant meal choices, this study aimed to verify the associations between food neophobia, familiarity with French cuisine, body mass, and French restaurant menu food choices in a sample of young Polish women.

## 2. Materials and Methods

### 2.1. Ethical Statement

The study was conducted on a sample of young Polish women who were not following a vegetarian diet of any kind. The sample was recruited from student and young social groups on social media based on an advertisement. The link for the qualification questionnaire was provided to all respondents, and if a potential respondent met the inclusion criteria, the main questionnaire was provided. Data for the study were collected using the Computer-Assisted Web Interview (CAWI) method.

The following inclusion criteria were taken into account:

- Women;
- Caucasian;
- Polish ethnicity;
- Age of 18–40 years; and
- Provided informed consent to participate.

The following exclusion criteria were taken into account:

- Pregnancy;
- Breastfeeding;
- Any diet-related disease;
- Any food allergy or intolerance;
- Following any other diet (e.g., vegetarian/vegan diet, low-calorie diet, etc.);
- Alcohol abstinence; and
- Any missing data within the food neophobia scale (FNS) questionnaire, familiarity with French cuisine, body weight and height, or model French restaurant menu questions.

No other criteria based on socio-economic status were taken into account, in order to include a wide range of participants representative of the general characteristics of young women in Poland. 

The total number of respondents meeting the inclusion criteria (*n* = 268) completed the questionnaire. Based on the exclusion criteria, *n* = 65 respondents were excluded due to pregnancy (*n* = 1), breastfeeding (*n* = 2), diet-related diseases (*n* = 4), food allergy or intolerance (*n* = 9), following other diets (*n* = 46), alcohol abstinence (*n* = 1), and missing data within the FNS questionnaire (*n* = 2). The final number of respondents participating in the study was *n* = 203.

The sample size was estimated based on the calculation for the population of Polish women aged 18–40 years (5,688,400, as reported by the Central Statistical Office (CSO) in Poland [24]), at a 95% confidence level and 10% margin of error while assuming a percentage of 50%. Taking into account the presented conditions, the required sample size was calculated as 96 respondents; thus, the recruited sample of 203 women was considered sufficient.

### 2.2. Food Choice Questionnaire

The food choice questionnaire was based on a model French restaurant menu developed exclusively for the study by a chef of Polish ethnicity, who was familiar with the characteristics of food neophobia, as well as with French cuisine. The prepared menu was later verified by a Polish nutritionist. Finally, the choice of products was discussed, and the menu was polished in such a way that it was appropriate for the study and understandable to Polish consumers (even those who were not familiar with French cuisine).

French cuisine is an important element of French culture [25], as it is associated with a food experience that is specific for France [26]. In Poland, French restaurants are quite common, and French cuisine is the second most popular European ethnic cuisine (after Italian cuisine) [27]. Moreover, it has been shown that the Polish culinary tradition has been influenced by French cuisine [28].

The model French restaurant menu was developed to include starters, soups, main courses, and desserts, with four dishes in each category. Dishes in each category had a similar energy value but differed in terms of (1) ingredients with neophobic potential for Polish consumers and (2) animal-based components. As animal-based components may induce a neophobic reaction, especially among women [29], the origin of ingredients was included as an additional variable. The neophobic potential for Polish consumers was determined based on the presence of ingredients not typical for Polish dishes (mussels, frogs), the presence of ingredients rejected by some Polish consumers (mushrooms, fish), or the use of ingredients that are not commonly used for a dish in Poland (broad bean soup, custard with champagne, white wine jelly). Dishes were planned using a 2 × 2 factorial design for the components with neophobic potential and animal-based components (four options of dishes: (1) no neophobic potential and animal-based components; (2) no neophobic potential and no animal-based components; (3) neophobic potential and animal-based components; and (4) neophobic potential and no animal-based components). Table 1 presents the dishes included in the model French restaurant menu developed for the study within a 2 × 2 factorial design for components of neophobic potential and animal-based components.

The respondents were informed that they would receive a menu of a French restaurant, without any additional information about the restaurant or its name. Each dish was presented on the menu with its French name and a simple description in Polish, without any additional information, such as price, list of ingredients, nutritional value, and photograph. The menu was prepared in an electronic version for each respondent separately, and a random order of dishes within each category was applied for each respondent.

The respondents were informed that they should imagine being in a French restaurant and receiving a menu to choose dishes that they would like to order. They were instructed to choose one dish from the category of starters, soups, main courses, and desserts (four dishes in total). They were informed that they should only consider their willingness to order specific dishes, and not other aspects (such as the supposed price of the dish, serving size, or nutritional value).

### 2.3. Studied Variables

The dependent variable within the study was the choice of dishes within the model French restaurant menu and their characteristics (in terms of the content of (1) the components of neophobic potential for Polish consumers and (2) animal-based components).

The independent variables included food neophobia, familiarity with French cuisine, and body mass.

Food neophobia was assessed while using the food neophobia scale (FNS) by Pliner and Hobden [12], which is a ten-item scale with five positive statements (confirmation indicates no food neophobic behaviors) and five negative statements (confirmation indicates food neophobic behaviors). The respondent is instructed to rate each statement in a seven-point Likert scale (from 1–strongly disagree to 7–strongly agree), and afterwards, the scores for positive statements are reversed. The final score on a scale from 10 to 70 is attributed to food neophobia level (based on the score) and food neophobia category (based on the terciles of the FNS score) [30].

In the studied group, the following terciles of the FNS score were defined:

- Low level of food neophobia: the first tercile of the FNS score (score of 10–24) (*n* = 68);
- Average level of food neophobia: the second tercile of the FNS score (score of 25–35) (*n* = 68);
- High level of food neophobia: the third tercile of the FNS score (score of 36–64) (*n* = 67).

Familiarity with French cuisine was assessed based on the one-item questionnaire on this topic (i.e., self-declared familiarity with French cuisine). The questionnaire included a question about the respondents’ familiarity with French cuisine and each respondent was instructed to indicate if they know French cuisine and its typical dishes (a closed-ended yes-no question). If the respondents did not know how to answer this question, they had the additional option to describe their familiarity with French cuisine (a descriptive answer to indicate known dishes and previous experiences with French cuisine). Afterwards, the descriptive answers were attributed to specific categories of familiarity with French cuisine and lack of familiarity with French cuisine. The simple definition was accepted, i.e., that familiarity with French cuisine must be based on a declared familiarity with at least three dishes and having consumed them at least once, whereas in the other cases (e.g., familiarity with one to two dishes, never consuming them, but only knowing their names), respondents were indicated as having no familiarity with French cuisine.

The body mass was assessed while using the body mass index, calculated on the basis of the standard equation of the World Health Organization with reference values of <18.5 kg/m^2^ for underweight, 18.5–25.0 kg/m^2^ for normal weight and >25.0 kg/m^2^ for overweight/obesity [31].

To characterize the studied group, additional questions were asked about place of residence (a closed-ended question with possible answers as follows: village; town/city of <500,000 residents; city of >500,000 residents); health status (a closed-ended question with possible answers as follows: very bad/bad; average; good/very good); diet quality (a closed-ended question with possible answers as follows: very bad/bad; average; good/very good), and economic status (a closed-ended question with possible answers as follows: very bad/bad; average; good/very good).

### 2.4. Statistical Analysis

The associations between independent variables (food neophobia, familiarity with French cuisine, and body mass) and dependent variables (choice of dishes within the model French restaurant menu and their characteristics) were studied.

The normality of distribution was studied while using the Shapiro–Wilk test. The internal consistency of the FNS was verified while using Cronbach’s alpha coefficient with the standard cutoff of 0.7, indicating good internal consistency [32]. Since the Cronbach’s alpha within the studied group was 0.76, a good internal consistency was confirmed.

The statistical analysis was conducted while using a chi^2^ test (to compare the share of respondents within sub-groups), as well as Student’s *t*-test and analysis of variance (ANOVA) (to compare the FNS results between groups, for parametric distributions), or the Mann–Whitney U test and Kruskal–Wallis ANOVA with a post-hoc Tukey test (to compare the FNS results between groups, for nonparametric distributions).

The statistical significance of differences was attributed to *p* ≤ 0.05. The statistical analysis was conducted while using Statistica version 13.3 (StatSoft Inc., Tulsa, OK, USA).

## 3. Results

### 3.1. Food Neophobia Results

The general characteristics of the studied sample of young Polish women is presented in Table 2. The majority of the studied respondents had normal body mass (71.4%), lived in towns or cities of <500,000 residents (44.3%) or of >500,000 residents (36.5%), and declared their diet quality as average (54.7%), their health status as good or very good (67.2%), and their economic status as good or very good (59.3%).

The FNS results in the studied sample of young Polish women are presented in Table 3. The median FNS score in the studied population of young Polish women was 31, differing from 10 to 64 (nonparametric distribution). For the low, average and high food neophobia levels, the median values of the FNS score were 19.5, 31.0, and 41.0, respectively.

### 3.2. Influence of Food Neophobia on French Menu Choices

The dish choices from the model French restaurant menu, stratified by food neophobia level, in the studied sample of young Polish women are presented in Table 4. In the conducted analysis, the food neophobia level was significantly associated with the dish choices within each meal category. For starters, respondents with a low food neophobia level chose moules à la marinière (mussels in white wine) more often (45.6%) than those with average (27.9%) and high levels (6.0%) of food neophobia. At the same time, respondents with a low level of food neophobia chose champignons farcis à la provencale (vegetable-stuffed champignon mushrooms) less often (29.4%) than those with average (42.6%) and high levels (58.2%) of food neophobia (*p* < 0.0001). For soups, respondents with low (48.5%) and average levels of food neophobia (57.4%) chose soupe à l’oignon (onion soup with toast) more often than those with a high level of food neophobia (35.8%). At the same time, respondents with low (10.3%) and average levels of food neophobia (20.6%) chose consommé (meat and vegetable broth) less often than those with a high level of food neophobia (52.2%) (*p* < 0.0001). For main courses, respondents with a low level of food neophobia chose cuisses de grenouille (frog legs) more often (27.9%) than those with average (13.2%) and high levels (3.0%) of food neophobia. At the same time, respondents with a low level of food neophobia chose ratatouille (vegetable stew) less often (16.2%) than those with average (33.8%) and high levels (47.8%) of food neophobia (*p* < 0.0001). For desserts, respondents with a high level of food neophobia chose salade de fruits de saison (seasonal fruit salad) more often (34.3%) than those with average (14.7%) and low levels (16.2%) of food neophobia. At the same time, respondents with a high level of food neophobia chose gelée de vin blanc aux fruits rouges (white wine jelly with red fruits) less often (7.5%) than those with average (17.6%) and low levels (26.5%) of food neophobia (*p* = 0.0139).

Table 5 presents the comparison of food neophobia levels among the studied sample of young Polish woman organized into sub-groups stratified according to the respondents’ choices of dishes from the model French restaurant menu. In the conducted analysis, the level of food neophobia was significantly associated with dish choices within each meal category. For starters, moules à la marinière (mussels in white wine) were chosen by the respondents with the lowest FNS score (lowest level of food neophobia) (*p* < 0.0001). For soups, consommé (meat and vegetable broth) was chosen by the respondents with the highest FNS score (highest level of food neophobia) (*p* < 0.0001). For main courses, cuisses de grenouille (frog legs) was chosen by the respondents with the lowest FNS score (lowest level of food neophobia), and ratatouille (vegetable stew) was chosen by the respondents with the highest FNS score (highest level of food neophobia) (*p* < 0.0001). For desserts, salade de fruits de saison (seasonal fruit salad) was chosen by the respondents with the highest FNS score (highest levels of food neophobia) (*p* < 0.0001).

Table 6 presents the dish choices that were made by the studied sample of young Polish women from the model French restaurant menu. These dishes are organized within sub-groups of dishes containing animal-based components/non-animal-based components, or those with non-neophobic components/with neophobic components, and are stratified by food neophobia level. For starters, respondents with a high level of food neophobia chose dishes with no animal-based components (*p* = 0.0004) more often (71.6%) than those with average (48.5%) and low levels (38.2%) of food neophobia. For soups, respondents with a high level of food neophobia chose dishes with animal-based components (*p* = 0.0027) more often (56.7%) than those with average (30.9%) and low levels (32.4%) of food neophobia; at the same time, they chose dishes with non-neophobic components more often (88.1%) than those with average (77.9%) and low levels (58.8%) of food neophobia (*p* = 0.0004). For main courses, respondents with a low level of food neophobia chose dishes with animal-based components more often (67.6%) than those with average (42.6%) and high levels (23.9%) of food neophobia (*p* < 0.0001). For desserts, respondents with a high level of food neophobia chose dishes with non-neophobic components more often (68.7%) than those with average (57.4%) and low levels (47.1%) of food neophobia (*p* = 0.0397).

Table 7 presents a comparison of the food neophobia level among the studied sample of young Polish women. The dishes from the model French restaurant menu are organized into different sub-groups and stratified according to those containing animal-based components/non-animal-based components, or those with non-neophobic components/with neophobic components. For starters, dishes with animal-based components were chosen by the respondents with a lower FNS score (lower level of food neophobia) (*p* < 0.0001). For soups, dishes with animal-based components were chosen by the respondents with higher FNS scores (higher levels of food neophobia) (*p* < 0.0001), whereas dishes with neophobic components were chosen by the respondents with a lower FNS score (lower level of food neophobia) (*p* = 0.0004). For main courses, dishes with animal-based components were chosen by the respondents with a lower FNS score (lower level of food neophobia) (*p* < 0.0001), and dishes with neophobic components were chosen by the respondents with a lower FNS score (lower level of food neophobia) (*p* = 0.0267). For desserts, dishes with neophobic components were chosen by the respondents with a lower FNS score (lower level of food neophobia) (*p* = 0.0010).

### 3.3. Influence of Familiarity with French Cuisine on French Menu Choices

Table 8 presents the dish choices made by the studied sample of young Polish women from the model French restaurant menu. These choices are stratified according to the respondents’ familiarity with French cuisine. In the conducted analysis, the familiarity with French cuisine was significantly associated with dish choices within the soup and main course categories. For soups, respondents familiar with French cuisine chose soupe à l’oignon (onion soup with toast) more often (66.7%) than those with no familiarity (43.5%) but chose consommé (meat and vegetable broth) less often (9.1% vs. 31.2%) (*p* = 0.0454). For main courses, respondents familiar with French cuisine chose cuisses de grenouille (frog legs) more often (39.4%) than those with no familiarity (13.5%), but chose duxelles (mushroom stew) less often (9.1% vs. 25.3%) (*p* = 0.0022).

Table 9 presents the dish choices made by the studied sample of young Polish women from the model French restaurant menu. The dishes are organized within sub-groups according to those containing animal-based components/non-animal-based components, or with non-neophobic components/with neophobic components, and are stratified according to the respondents’ familiarity with French cuisine. For starters, respondents familiar with French cuisine chose dishes with animal-based components more often (63.6%) than those with no familiarity (44.1%) (*p* = 0.0399). For soups, respondents familiar with French cuisine chose dishes with animal-based components less often (21.2%) than those with no familiarity (43.5%) (*p* = 0.0166). For main courses, respondents familiar with French cuisine chose dishes with animal-based components more often (69.7%) than those with no familiarity (43.5%) (*p* = 0.0059).

### 3.4. Influence of Body Mass on French Menu Choices

Table 10 presents the dish choices made by the studied sample of young Polish women from the model French restaurant menu, which are stratified according to body mass. In the conducted analysis, the body mass was not associated with dish choices from the model French restaurant menu.

Table 11 presents the dish choices made by the studied sample of young Polish women from the model French restaurant menu. The dishes are organized within sub-groups of those containing animal-based components/non-animal-based components, or those with non-neophobic components/with neophobic components. In the conducted analysis, body mass was not associated with dish choices from the model French restaurant menu.

## 4. Discussion

The statistical analysis performed in the study indicated that food neophobia and familiarity with French cuisine were associated with food choices from a French restaurant menu, but not with body mass. The influence of food neophobia and familiarity with the cuisine on food choice may be attributed to the specific model applied in the study, as it was not conducted on dishes of a known cuisine, but rather an ethnic European cuisine which was unknown to a majority of respondents (80% of the studied group declared it to be unfamiliar). In such cases, the role of body mass on food choice may be negligible, whereas food neophobia and cuisine familiarity may be the major determinants of food choice.

In general, body mass may be associated with nutritional behaviors. It has been shown that body mass can also be linked with food choice determinants [33] and dietary patterns [34]. Similarly, body mass is associated with emotional eating behavior, which is defined as the tendency to eat energy-dense products in response to negative emotions [35]. Moreover, body mass may be specifically associated with food choices, including the choices of restaurant dishes. Excessive body mass is caused by the overconsumption of ready-to-eat food products [36] and eating in fast-food restaurants [37]. In addition, it is associated with higher portion sizes of energy-dense products [38]. This may be related to the influence of food product choice on body mass, as the excessive availability of convenience stores increases the risk of higher BMI [39]. However, the reverse causality cannot be ruled out, as body mass may also be associated with acceptance, attitude, and motivation toward body mass reduction [40], which may consequently influence the diet followed by an individual [41].

As described above, a general association can be found between body mass and food choice, but such a relationship was not observed in the studied group. This may be due to the fact that the study did not analyze the general food choice, but the food choice within a French restaurant menu, which may have been experienced by the studied group only theoretically and once in a lifetime. At the same time, the lack of an association with BMI may have been influenced by more powerful factors, namely food neophobia and familiarity with French cuisine, which were the major food choice determinants in this study.

In the studied group, food neophobia was an important determinant of food choice, as respondents with a high level of food neophobia chose dishes with neophobic components, which was observed for soups and desserts, less often than those with a low level of food neophobia. In the absence of such an association, respondents with a high level of food neophobia chose dishes with animal-based components, which was observed for starters and main courses, less often than those with a low level of food neophobia. The general association between food neophobia and the choice of dishes with components of non-neophobic potential (familiar to the studied sample) was also stated in previous studies [16,17]. Similarly, associations between food neophobia and the reduced intake of fruits and vegetables [42], as well as low diet variety, have been well established [43]. However, in the present study, apart from neophobic potential, another important characteristic that prevented food neophobic individuals from choosing a dish was the presence of animal-based ingredients. Animal-based components may be associated with the avoidance of a product by food neophobic individuals, as it may be related to food disgust due to a high risk of the transmission of toxins and pathogens, as well as a need to protect social order and follow moral principles [44]. Numerous studies have shown that food neophobia was associated with the reduced consumption or pleasure from the consumption of meat and offal [14,45,46], as well as fish and shellfish [14,46,47]. Therefore, it may be indicated that in the studied group, the major motivator for food neophobic individuals to avoid unknown/animal-based products and choose other products was either no familiarity with the components of a dish or the presence of animal-based components. Such behavior may induce reduced dietary diversity, as a result of choosing only well-known food products, or products with no animal-based components [48].

At the same time, in the present study, respondents who were familiar with French cuisine chose dishes with animal-based components more often, which was observed for starters and desserts, compared to those with no familiarity, but a reverse association was observed for soups. The fact that a dish originates from another culture is the major reason for the negative attitude of food neophobic individuals toward this dish [46]. Therefore, it may be indicated that familiarity with a specific cuisine may change the food choices within a restaurant menu. For starters and desserts, familiarity with French cuisine encouraged respondents to choose dishes containing animal-based components, which may be associated with reduced disgust [44]. However, a reverse association was observed for soups, which may have resulted from the description of an animal-based soup with non-neophobic potential, namely consommé, as a meat and vegetable broth. For Poles, this description may resemble one of the most known Polish soups, namely Rosół, which is a meat and vegetable broth prepared most often from chicken and served with capellini pasta [49]. Therefore, even for respondents with no familiarity with French cuisine, the description of consommé may have sounded familiar and encouraged them to choose this soup.

The present study indicated that food neophobia and cuisine familiarity influenced the choice of dishes from a French restaurant menu. Although the study revealed some interesting findings, further research is needed and similar studies should be conducted on vegetarian/vegan populations, using the model French restaurant menu, including dishes with non-animal-based components. In addition, dietary diversity should be studied. Despite providing valuable findings, the study had certain limitations such as a small sample size, self-reported restaurant choices, and the inclusion of only young women.

## 5. Conclusions

It may be concluded that food neophobia and familiarity with French cuisine may be important determinants of dish choices from a French restaurant menu. No influence of body mass on the choice of dishes from the model French restaurant menu was stated. The presence of unfamiliar ingredients and of animal-based ingredients may reduce the frequency with which specific dishes from a French restaurant menu are chosen, which may result in a reduced diversity in individuals’ diets.

## Figures and Tables

**Table 1:** The dishes included in the model French restaurant menu developed for the study, within a 2 × 2 factorial design for components of neophobic potential and animal-based components.

Meal	Name of the Dish	Simple Description of the Dish Presented within the Menu	Components of Neophobic Potential for Polish Consumers	Animal-Based Components
Starter	Quiche Lorraine	Tart with bacon	-	Bacon
	Salade de betteraves	Beetroot salad	-	-
	Moules à la marinière	Mussels in white wine	Mussels	Mussels
	Champignons farcis à la provencale	Vegetable-stuffed champignon mushrooms	Champignon mushrooms	-
Soup	Consommé	Meat and vegetable broth	-	Meat
	Soupe à l’oignon	Onion soup with toast	-	-
	Bouillabaisse	Fish soup	Fish	Fish
	Soupe aux fèves	Broad bean soup	Broad bean soup	-
Main course	Boeuf Bourguignon	Burgundy-style beef stew	-	Beef
	Ratatouille	Vegetable stew	-	-
	Cuisses de grenouille	Frog legs	Frog	Frog
	Duxelles	Mushroom stew	Mushroom	-
Dessert	Crème brûlée	Cream and egg-based vanilla pudding	-	Cream and eggs
	Salade de fruits de saison	Seasonal fruit salad	-	-
	Champagne Sabayon	Champagne-based egg custard	Custard with champagne	Eggs
	Gelée de vin blanc aux fruits rouges	White wine jelly with red fruits	White wine jelly	-

**Table 2:** The general characteristics of the studied sample of young Polish women.

Characteristics		Values
Age (years)	Mean ± SD	24.6 ± 3.5
	Median (25th–75th)	24.0 * (23.0–25.0)
Body mass (assesssed based on BMI)	Underweight	20 (9.9%)
	Normal	145 (71.4%)
	Overweight/obese	38 (18.7%)
Residence	Village	39 (19.2%)
	Towns and cities of <500,000 residents	90 (44.3%)
	Cities of >500,000 residents	74 (36.5%)
Declared diet quality	Very bad or bad	11 (5.4%)
	Average	111 (54.7%)
	Good or very good	79 (38.9%)
	No answer	2 (1.0%)
Declared health status	Very bad or bad	8 (4.1%)
	Average	56 (28.7%)
	Good or very good	131 (67.2%)
Declared economic status	Very bad or bad	8 (4.5%)
	Average	63 (35.6%)
	Good or very good	105 (59.3%)
	No answer	1 (0.6%)

* Nonparametric distribution (verified by the Shapiro–Wilk test; *p* ≤0.05); BMI–body mass index.

**Table 3:** The food neophobia scale (FNS) results in the studied sample of young Polish women.

FNS Score	Total (*n* = 203)	Food Neophobia Level **		
		Low (*n* = 68)	Average (*n* = 68)	High (*n* = 67)
Mean ± SD	30.6 ± 10.4	19.3 ± 3.5	30.2 ± 3.0	42.6 ± 5.5
95% CI	29.2–32.1	18.5–20.2	29.5–30.9	41.3–44.0
Median	31.0 *	19.5 *	31.0 *	41.0 *
Range	10–64	10–24	25–35	36–64
25th–75th	22–39	17.5–22	28–33	39–46

* Nonparametric distribution (verified by the Shapiro–Wilk test; *p* ≤ 0.05); ** low, average and high food neophobia levels attributed to the first (score of 10–24), second (score of 25–35) and third terciles of the FNS score (score of 36–64); CI–confidence interval.

**Table 4:** The dish choices made by the studied sample of young Polish women from the model French restaurant menu, stratified by the level of food neophobia.

Meal	Dish	Total (*n* = 203)	Food Neophobia Level *—*n* (%)			*p*-Value **
			Low (*n* = 68)	Average (*n* = 68)	High (*n* = 67)	
Starters	Quiche Lorraine	42 (20.7%)	11 (16.2%)	16 (23.5%)	15 (22.4%)	<0.0001
	Salade de betteraves	19 (9.4%)	6 (8.8%)	4 (5.9%)	9 (13.4%)	
	Moules à la marinière	54 (26.6%)	31 (45.6%)	19 (27.9%)	4 (6.0%)	
	Champignons farcis à la provencale	88 (43.3%)	20 (29.4%)	29 (42.6%)	39 (58.2%)	
Soup	Consommé	56 (27.6%)	7 (10.3%)	14 (20.6%)	35 (52.2%)	<0.0001
	Soupe à l’oignon	96 (47.3%)	33 (48.5%)	39 (57.4%)	24 (35.8%)	
	Bouillabaisse	25 (12.3%)	15 (22.1%)	7 (10.3%)	3 (4.5%)	
	Soupe aux fèves	26 (12.8%)	13 (19.1%)	8 (11.8%)	5 (7.5%)	
Main course	Boeuf Bourguignon	61 (30.0%)	27 (39.7%)	20 (29.4%)	14 (20.9%)	<0.0001
	Ratatouille	66 (32.5%)	11 (16.2%)	23 (33.8%)	32 (47.8%)	
	Cuisses de grenouille	30 (14.8%)	19 (27.9%)	9 (13.2%)	2 (3.0%)	
	Duxelles	46 (22.7%)	11 (16.2%)	16 (23.5%)	19 (28.4%)	
Dessert	Crème brûlée	73 (36.0%)	21 (30.9%)	29 (42.6%)	23 (34.3%)	0.0139
	Salade de fruits de saison	44 (21.7%)	11 (16.2%)	10 (14.7%)	23 (34.3%)	
	Champagne Sabayon	51 (25.1%)	18 (26.5%)	17 (25.0%)	16 (23.9%)	
	Gelée de vin blanc aux fruits rouges	35 (17.2%)	18 (26.5%)	12 (17.6%)	5 (7.5%)	

* Low, average and high levels of food neophobia attributed to the first (score of 10–24), second (score of 25–35) and third terciles of the FNS score (score of 36–64); ** chi^2^ test.

**Table 5:** The comparison of food neophobia levels among the studied sample of young Polish women organized into sub-groups stratified according to dish choices from the model French restaurant menu.

Meal	Dish	Mean FNS	Median (Min–Max)	*p*-Value **
Starters	Quiche Lorraine	31.3 ± 9.9	31.5 (15–51) ^a^	<0.0001
	Salade de betteraves	34.3 ± 11.9	33.0 (15–56) ^a^	
	Moules à la marinière	24.1 ± 7.9	23.5 (10–48) ^b^	
	Champignons farcis à la provencale	33.6 ± 10.1	34 (14–64) ^a^	
Soup	Consommé	37.1 ± 9.5	38.5 (18–64) ^a^	<0.0001
	Soupe à l’oignon	29.3 ± 9.8	28 (11–59) ^b^	
	Bouillabaisse	25.1 ± 9.4	22.5 (10–48) ^b^	
	Soupe aux fèves	27.4 ± 9.2	26 (10–45) ^b^	
Main course	Boeuf Bourguignon	28.2 ± 9.1	27 * (12–49) ^ab^	<0.0001
	Ratatouille	35.3 ± 10.0	34 (17–64) ^c^	
	Cuisses de grenouille	22.7 ± 8.9	21 (10–45) ^b^	
	Duxelles	32.4 ± 9.8	32.5 (15–59) ^ac^	
Dessert	Crème brûlée	30.8 ± 8.7	31 (12–50) ^ab^	<0.0001
	Salade de fruits de saison	36.2 ± 12.1	36 (16–64) ^b^	
	Champagne Sabayon	29.3 ± 9.6	29 (10–48) ^a^	
	Gelée de vin blanc aux fruits rouges	25.4 ± 9.4	24 (10–49) ^a^	

* Nonparametric distribution (verified by the Shapiro–Wilk test; *p* ≤ 0.05); ** analysis of variance (ANOVA)/ Kruskal–Wallis ANOVA with a post-hoc Tukey test (based on distribution); a, b, c in the superscript are attributed to statistically significant differences.

**Table 6:** The dish choices made by the studied sample of young Polish women from the model French restaurant menu. The dishes are organized within sub-groups of dishes containing animal-based components/non-animal-based components, or with non-neophobic components/with neophobic components.

Meal	Dish	Food Neophobia Level *—*n* (%)			*p*-Value **
		Low (*n* = 67)	Average (*n* = 68)	High (*n* = 68)	
Starters	Animal-based components	42 (61.8%)	35 (51.5%)	19 (28.4%)	0.0004
	Non-animal-based components	26 (38.2%)	33 (48.5%)	48 (71.6%)	
	With non-neophobic components	17 (25.0%)	20 (29.4%)	24 (35.8%)	0.3867
	With neophobic components	51 (75.0%)	48 (70.6%)	43 (64.2%)	
Soup	Animal-based components	22 (32.4%)	21 (30.9%)	38 (56.7%)	0.0027
	Non-animal-based components	46 (67.6%)	47 (69.1%)	29 (43.3%)	
	With non-neophobic components	40 (58.8%)	53 (77.9%)	59 (88.1%)	0.0004
	With neophobic components	28 (41.2%)	15 (22.1%)	8 (11.9%)	
Main course	Animal-based components	46 (67.6%)	29 (42.6%)	16 (23.9%)	<0.0001
	Non-animal-based components	22 (32.4%)	39 (57.4%)	51 (76.1%)	
	With non-neophobic components	38 (55.9%)	43 (63.2%)	46 (68.7%)	0.3056
	With neophobic components	30 (44.1%)	25 (36.8%)	21 (31.3%)	
Dessert	Animal-based components	39 (57.4%)	46 (67.6%)	39 (58.2%)	0.3940
	Non-animal-based components	29 (42.6%)	22 (32.4%)	28 (41.8%)	
	With non-neophobic components	32 (47.1%)	39 (57.4%)	46 (68.7%)	0.0397
	With neophobic components	36 (52.9%)	29 (42.6%)	21 (31.3%)	

* Low, average and high levels of food neophobia attributed to the first (score of 10–24), second (score of 25–35) and third tercile of the FNS score (score of 36–64); ** chi^2^ test.

**Table 7:** The comparison of the food neophobia level among the studied sample of young Polish women. The dishes from the model French restaurant menu are organized into sub-groups and the food neophobia level of the dishes is stratified according to whether they contain animal-based components/non-animal-based components, or non-neophobic components/with neophobic components.

Meal	Dish	Mean FNS	Median (Min–Max)	*p*-Value **
Starters	Animal-based components	27.2 ± 9.3	29 (20–51)	<0.0001
	Non-animal-based components	33.7 ± 10.4	34 (14–64)	
	With non-neophobic components	32.3 ± 10.3	32 (15–54)	0.1499
	With neophobic components	30 ± 10.4	29.5 * (10–64)	
Soup	Animal-based components	33.4 ± 10.9	34 (10–64)	<0.0001
	Non-animal-based components	28.9 ± 9.7	28 * (10–59)	
	With non-neophobic components	32.1 ± 10.4	32 (11–64)	0.0004
	With neophobic components	26.3 ± 9.2	23 (10–48)	
Main course	Animal-based components	26.4 ± 9.3	24 (10–49)	<0.0001
	Non-animal-based components	34.2 ± 10.0	33.5 * (15–64)	
	With non-neophobic components	31.9 ± 10.2	31 * (12–64)	0.0267
	With neophobic components	28.6 ± 10.5	28.5 (10–59)	
Dessert	Animal-based components	30.2 ± 9.1	30 (10–50)	0.7528
	Non-animal-based components	31.4 ± 12.2	31 * (10–64)	
	With non-neophobic components	32.8 ± 10.4	32 * (12–64)	0.0010
	With neophobic components	27.7 ± 9.7	27 * (10–49)	

* Nonparametric distribution (verified by the Shapiro–Wilk test; *p* ≤0.05); ** Student’s t-test/ Mann–Whitney U test (based on distribution).

**Table 8:** The dish choices made by the studied sample of young Polish women from the model French restaurant menu, stratified according to their familiarity with French cuisine.

Meal	Dish	Familiarity with French Cuisine		*p*-Value *
		Familiar (*n* = 33)	Unfamiliar (*n* = 170)	
Starters	Quiche Lorraine	9 (27.3%)	33 (19.4%)	0.2364
	Salade de betteraves	2 (6.1%)	17 (10.0%)	
	Moules à la marinière	12 (36.4%)	42 (24.7%)	
	Champignons farcis à la provencale	10 (30.3%)	78 (45.9%)	
Soup	Consommé	3 (9.1%)	53 (31.2%)	0.0454
	Soupe à l’oignon	22 (66.7%)	74 (43.5%)	
	Bouillabaisse	4 (12.1%)	21 (12.4%)	
	Soupe aux fèves	4 (12.1%)	22 (12.9%)	
Main course	Boeuf Bourguignon	10 (30.3%)	51 (30.0%)	0.0022
	Ratatouille	7 (21.2%)	53 (31.2%)	
	Cuisses de grenouille	13 (39.4%)	23 (13.5%)	
	Duxelles	3 (9.1%)	43 (25.3%)	
Dessert	Crème brûlée	14 (42.4%)	59 (34.7%)	0.2874
	Salade de fruits de saison	3 (9.1%)	41 (24.1%)	
	Champagne Sabayon	9 (27.3%)	42 (24.7%)	
	Gelée de vin blanc aux fruits rouges	7 (21.2%)	28 (16.5%)	

* chi^2^ test.

**Table 9:** The dish choices made by the studied sample of young Polish women from the model French restaurant menu. The dishes are organized within sub-groups of those containing animal-based components/non-animal-based components, or those with non-neophobic components/with neophobic components, and are stratified according to the respondents’ familiarity with French cuisine.

Meal	Dish	Familiarity with French Cuisine		*p*-Value *
		Familiar (*n* = 33)	Unfamiliar (*n* = 170)	
Starters	Animal-based components	21 (63.6%)	75 (44.1%)	0.0399
	Non-animal-based components	12 (36.4%)	95 (55.9%)	
	With non-neophobic components	11 (33.3%)	50 (29.4%)	0.6531
	With neophobic components	22 (66.7%)	120 (70.6%)	
Soup	Animal-based components	7 (21.2%)	74 (43.5%)	0.0166
	Non-animal-based components	26 (78.8%)	96 (56.5%)	
	With non-neophobic components	25 (75.8%)	127 (74.7%)	0.8993
	With neophobic components	8 (24.2%)	43 (25.3%)	
Main course	Animal-based components	23 (69.7%)	74 (43.5%)	0.0059
	Non-animal-based components	10 (30.3%)	96 (56.5%)	
	With non-neophobic components	17 (51.5%)	104 (61.2%)	0.3007
	With neophobic components	16 (48.5%)	66 (38.8%)	
Dessert	Animal-based components	23 (69.7%)	101 (59.4%)	0.2674
	Non-animal-based components	10 (30.3%)	69 (40.6%)	
	With non-neophobic components	17 (51.5%)	100 (58.8%)	0.4367
	With neophobic components	16 (48.5%)	70 (41.2%)	

* chi^2^ test.

**Table 10:** The dish choices from the model French restaurant menu, stratified by body mass, in the studied sample of young Polish women.

Meal	Dish	Body Mass (Assessed Based on BMI) *			*p*-Value **
		Underweight (*n* = 20)	Underweight (*n* = 20)	Underweight (*n* = 20)	
Starters	Quiche Lorraine	6 (30%)	29 (20%)	7 (18.4%)	0.6430
	Salade de betteraves	2 (10%)	15 (10.3%)	2 (5.3%)	
	Moules à la marinière	3 (15%)	42 (29%)	9 (23.7%)	
	Champignons farcis à la provencale	9 (45%)	59 (40.7%)	20 (52.6%)	
Soup	Consommé	5 (25%)	43 (29.7%)	8 (21.1%)	0.4361
	Soupe à l'oignon	11 (55%)	68 (46.9%)	17 (44.7%)	
	Bouillabaisse	1 (5%)	17 (11.7%)	7 (18.4%)	
	Soupe aux fèves	3 (15%)	17 (11.7%)	6 (15.8%)	
Main course	Boeuf Bourguignon	7 (35%)	42 (29%)	12 (31.6%)	0.2900
	Ratatouille	4 (20%)	50 (34.5%)	12 (31.6%)	
	Cuisses de grenouille	1 (5%)	25 (17.2%)	4 (10.5%)	
	Duxelles	8 (40%)	28 (19.3%)	10 (26.3%)	
Dessert	Crème brûlée	8 (40%)	51 (35.2%)	14 (36.8%)	0.8855
	Salade de fruits de saison	3 (15%)	31 (21.4%)	10 (26.3%)	
	Champagne Sabayon	4 (20%)	39 (26.9%)	8 (21.1%)	
	Gelée de vin blanc aux fruits rouges	5 (25%)	24 (16.6%)	6 (15.8%)	

* Underweight, normal body mass and overweight/obesity attributed to the BMI of <18.5 kg/m^2^ for underweight, 18.5–25.0 kg/m^2^ for normal weight and >25.0 kg/m^2^ for overweight/obesity; ** chi^2^ test; BMI–body mass index.

**Table 11:** The dish choices made by the studied sample of young Polish women from the model French restaurant menu. The dishes are organized within sub-groups of those containing animal-based components/non-animal-based components, or those with non-neophobic components/with neophobic components.

Meal	Dish	Body Mass (Assessed Based on BMI) *			*p*-Value **
		Underweight (*n* = 20)	Normal (*n* = 145)	Overweight/Obese (*n* = 38)	
Starters	Animal-based components	9 (45.0%)	71 (49.0%)	16 (42.1%)	0.7353
	Non-animal-based components	11 (55.0%)	74 (51.0%)	22 (57.9%)	
	With non-neophobic components	8 (40.0%)	44 (30.3%)	9 (23.7%)	0.4315
	With neophobic components	12 (60.0%)	101 (69.7%)	29 (76.3%)	
Soup	Animal-based components	6 (30.0%)	60 (41.4%)	15 (39.5%)	0.6210
	Non-animal-based components	14 (70.0%)	85 (58.6%)	23 (60.5%)	
	With non-neophobic components	16 (80.0%)	111 (76.6%)	25 (65.8%)	0.3391
	With neophobic components	4 (20.0%)	34 (23.4%)	13 (34.2%)	
Main course	Animal-based components	8 (40.0%)	67 (46.2%)	16 (42.1%)	0.8130
	Non-animal-based components	12 (60.0%)	78 (53.8%)	22 (57.9%)	
	With non-neophobic components	11 (55.0%)	92 (63.4%)	24 (63.2%)	0.7622
	With neophobic components	9 (45.0%)	53 (36.6%)	14 (36.8%)	
Dessert	Animal-based components	12 (60.0%)	90 (62.1%)	22 (57.9%)	0.8910
	Non-animal-based components	8 (40.0%)	55 (37.9%)	16 (42.1%)	
	With non-neophobic components	11 (55.0%)	82 (56.6%)	24 (63.2%)	0.7404
	With neophobic components	9 (45.0%)	63 (43.4%)	14 (36.8%)	

* Underweight, normal body mass and overweight/obesity attributed to the BMI of <18.5 kg/m^2^ for underweight, 18.5–25.0 kg/m^2^ for normal weight and >25.0 kg/m^2^ for overweight/obesity; ** chi^2^ test; BMI–body mass index.
